# *International Journal of Molecular Sciences* 2016 Best Paper Award

**DOI:** 10.3390/ijms17050777

**Published:** 2016-05-20

**Authors:** International Journal of Molecular Sciences Editorial Office

**Affiliations:** MDPI AG, Klybeckstrasse 64, CH-4057 Basel, Switzerland; ijms@mdpi.com

The Editors of the *International Journal of Molecular Sciences* have established the Best Paper Award to recognize the most outstanding articles published in the areas of molecular biology, molecular physics and chemistry that have been published in the *International Journal of Molecular Sciences*. The prizes have been awarded annually since 2012 [1,2,3,4].

We are pleased to announce the winners of the “*International Journal of Molecular Sciences* Best Paper Award” for 2016. Nominations were selected by the Editorial Board from all papers published in 2015. The awards are issued to reviews and research articles separately. Following extensive review by the Editorial Board, five top-voted research articles and the three top-voted reviews have been awarded the “*International Journal of Molecular Sciences* Best Paper Award” for 2016. The papers are listed as follows, and in no particular order:

**Original Research Article Award:**
  - **A Variant in the Osteoprotegerin Gene Is Associated with Coronary Atherosclerosis in Patients with Rheumatoid Arthritis: Results from a Candidate Gene Study**
    Cecilia P. Chung, Joseph F. Solus, Annette Oeser, Chun Li, Paolo Raggi, Jeffrey R. Smith and C. Michael Stein
    *Int. J. Mol. Sci.* **2015**, *16*(2), 3885–3894; doi:10.3390/ijms16023885
    Available online: http://www.mdpi.com/1422-0067/16/2/3885

*Awarding Committee Comments*
  “This article addresses the relevant issue of increased atherosclerosis (and cardiovascular risk) in patients with rheumatoid arthritis. Identification of a polymorphism in gene encoding osteoprotegerin (involved in bone metabolism and associated with atherosclerosis) and, above all, its association with atherosclerosis in these patients, highlight novel mechanistic findings in this inflammatory disease.”
  *By Dr. Marcello Iriti*
  “A variant in OPG gene is associated with coronary atherosclerosis…This paper is a good example of the use of applied statistics to identify risk factors in a relevant patient population, and should serve as a benchmark for studies employing similar approaches.”
  *By Dr. Joseph V. Moxon*
  - **Ameliorative Effects of PACAP against Cartilage Degeneration. Morphological, Immunohistochemical and Biochemical Evidence from *in Vivo* and *in Vitro* Models of Rat Osteoarthritis**
    Salvatore Giunta, Alessandro Castorina, Rubina Marzagalli, Marta Anna Szychlinska, Karin Pichler, Ali Mobasheri and Giuseppe Musumeci
    *Int. J. Mol. Sci.* **2015**, *16*(3), 5922–5944; doi:10.3390/ijms16035922
    Available online: http://www.mdpi.com/1422-0067/16/3/5922

*Awarding Committee Comments:*
  “Authors describe an anti-inflammatory and antiapoptotic chondroprotective factor in an elegant in vivo/in vitro model of osteoarthritis. This suggests a novel therapeutic approach in joint inflammation, as well as an activator of cartilage regeneration in degenerative diseases.”
  *By Dr. Marcello Iriti*
  “A nice study with both in vivo and in vitro data to demonstrate the association and protective effect of the neuropeptide PACAP in osteoarthritis.”
  *By Dr. Vera Sau-Fong Chan*
  - **Cell Adhesion and *in Vivo* Osseointegration of Sandblasted/Acid Etched/Anodized Dental Implants**
    Mu-Hyon Kim, Kyeongsoon Park, Kyung-Hee Choi, Soo-Hong Kim, Se Eun Kim, Chang-Mo Jeong and Jung-Bo Huh
    *Int. J. Mol. Sci.* **2015**, *16*(5), 10324–10336; doi:10.3390/ijms160510324
    Available online: http://www.mdpi.com/1422-0067/16/5/10324

*Awarding Committee Comments:*
  “A new type of surface modified titanium dental implant improved osseointegration in an in vitro/in vivo model, i.e., increased cell adhesion and bone-to-implant contact. This paper will certainly be of interest in the field of implant dentistry and dental sciences.”
  *By Dr. Marcello Iriti*
  “This paper presents findings to show the enhanced biocompatibility of application of the potential action of a new type of surface-modified titanium material for dental implant, revealing its potential translational impact.”
  *By Dr. Vera Sau-Fong Chan*
  - **Dimethyl Fumarate Protects Neural Stem/Progenitor Cells and Neurons from Oxidative Damage through Nrf2-ERK1/2 MAPK Pathway**
    Qin Wang, Sergei Chuikov, Sophina Taitano, Qi Wu, Arjun Rastogi, Samuel J. Tuck, Joseph M. Corey, Steven K. Lundy and Yang Mao-Draayer
    *Int. J. Mol. Sci.* **2015**, *16*(6), 13885–13907; doi:10.3390/ijms160613885
    Available online: http://www.mdpi.com/1422-0067/16/6/13885

*Awarding Committee Comments:*
  “A quite sound paper about the role of dimethyl fumarate-induced Nrf2 pathway in neuronal progenitor cells maintenance and protection against oxidative stress. Such results may certainly contribute to a better targeting of Multiple Sclerosis-associated signaling pathways.”
  *By Dr. Anthony Lemarié*
  “A very interesting mechanistic study focusing on dimethyl fumarate effects in multiple sclerosis. This drug reduces oxidative stress in neural stem/progenitor cells, increasing the expression of neuroprotective transcription factors at both levels of RNA and protein, and upregulating anti-oxidative stress genes. This paper suggests further insights into new targets for treatment of this chronic neurological disease affecting young adults.”
  *By Dr. Marcello Iriti*
  “The authors showed through a well constructed set of experiments that dimethyl fumarate conferred protection to neural stem/progenitor cells from oxidative damage. The findings of this study will form the basis of new experiments that will enhance our understanding of multiple sclerosis and assist in development treatments that will be of benefit to people who suffer from this condition.”
  *By Dr. Terrence Piva*
  - **Integrated Bioinformatics, Environmental Epidemiologic and Genomic Approaches to Identify Environmental and Molecular Links between Endometriosis and Breast Cancer**
    Deodutta Roy, Marisa Morgan, Changwon Yoo, Alok Deoraj, Sandhya Roy, Vijay Kumar Yadav, Mohannad Garoub, Hamza Assaggaf and Mayur Doke
    *Int. J. Mol. Sci.* **2015**, *16*(10), 25285–25322; doi:10.3390/ijms161025285
    Available online: http://www.mdpi.com/1422-0067/16/10/25285

*Awarding Committee Comments:*
  “The authors integrate, in a unique way, information from epidemiology, genomics and bioinformatics, thereby significantly advancing our understanding on the links between human disease and exposure to endocrine disrupting chemicals.”
  *By Dr. Helmut Segner*
  “Integrated bioinformatics, environmental epidemiologic and genomic approaches...An innovative approach allowing researchers to mine previously published data, consolidate existing knowledge, and synthesise new hypotheses.”
  *By Dr. Joseph V. Moxon*
  “A really comprehensive integrated bioinformatics (with environmental data, epidemiology and genomics) approach identified common molecular traits in breast cancer and endometriosis caused by exposure to endocrine disruptors, i.e., altered environmentally and estrogen-responsive genes in both conditions. A great example of gene–environment interaction in cancer development.”
  *By Dr. Marcello Iriti*
  “Through the use of environmental epidemiologic, genomic, and bioinformatics the authors showed that exposure to polychlorinated biphenyls (PCBs) increase an individuals risk factor of breast cancer or endometriosis, and they share some common environmental and molecular risk factors. This was a well performed study and has yielded some interesting results which will help us further understand how PCBs exert their detrimental effects on individuals, which will assist in the development of treatments which can confer protection to their actions.”
  *By Dr. Terrence Piva*
  “An interesting analysis using public databases and bioinformatics tools to reveal common environmental and molecular risk factors between two estrogen-sensitive conditions.”
  *By Dr. Vera Sau-Fong Chan*

**Review Paper Award:**
  - **Starting to Gel: How Arabidopsis Seed Coat Epidermal Cells Produce Specialized Secondary Cell Walls**
    Cătălin Voiniciuc, Bo Yang, Maximilian Heinrich-Wilhelm Schmidt, Markus Günl and Björn Usadel
    *Int. J. Mol. Sci.* **2015**, *16*(2), 3452–3473; doi:10.3390/ijms16023452
    Available online: http://www.mdpi.com/1422-0067/16/2/3452

*Awarding Committee Comments:*
  “As Editor of this article, I previously appreciated it, a piece of cell wall research. The seed coat epidermis provides an alternative system to investigate the production of cell wall polysaccharides, particularly the synthesis and modification of pectin, thus improving our understanding of plant cell walls.”
  *By Dr. Marcello Iriti*
  “A structured review on a fascinating topic.”
  *By Dr. Vera Sau-Fong Chan*
  - **Sequencing Overview of Ewing Sarcoma: A Journey across Genomic, Epigenomic and Transcriptomic Landscapes**
    Laurens G. L. Sand, Karoly Szuhai and Pancras C. W. Hogendoorn
    *Int. J. Mol. Sci.* **2015**, *16*(7), 16176–16215; doi:10.3390/ijms160716176
    Available online: http://www.mdpi.com/1422-0067/16/7/16176

*Awarding Committee Comments:*
  “A really OMICS approach! By sequencing Ewing sarcoma at the genome, epigenome and transcriptome level, novel predictive markers and candidates for immuno- and targeted-therapy in young patients can be identified, as summarized in this review.”
  *By Dr. Marcello Iriti*
  “A very comprehensive and informative review with a nice touch on implications in targeted therapy—a good read to gain understanding in Ewing Sarcoma genomics.”
  *By Dr. Vera Sau-Fong Chan*
  - **Long Non-Coding RNAs in Endometrial Carcinoma**
    Maria A. Smolle, Marc D. Bullock, Hui Ling, Martin Pichler and Johannes Haybaeck
    *Int. J. Mol. Sci.* **2015**, *16*(11), 26463–26472; doi:10.3390/ijms161125962
    Available online: http://www.mdpi.com/1422-0067/16/11/25962

*Awarding Committee Comments:*
  “A very novel topic: lncRNAs (long non-coding RNAs)! Specific and altered expression patterns of lncRNAs in endometrial carcinoma compared to normal endometrial tissues are reviewed, as well as the potential of deregulated lncRNAs as biomarkers. In fact, a number of lncRNAs are differentially expressed in normal, hyperplastic and dysplastic endometrium which can be targeted in order to achieve anticancer activity.”
  *By Dr. Marcello Iriti*
  “The authors reviewed the role different long non-coding RNAs (lncRNAs) play in the development of different types of endometrial carcinomas (EC). The review was well written and discussed the two major forms of EC and as such will form a useful reference for those studying the role of lncRNAs in the development of different endometrial carcinomas.”
  *By Dr. Terrence Piva*
  “Understanding lncRNAs in cancer biology is one of the hottest research areas—this article provides a timely review on recent development in endometrial carcinoma.”
  *By Dr. Vera Sau-Fong Chan*

These eight exceptional papers represent valuable contributions to the *International Journal of Molecular Sciences* and their respective scientific research fields. On behalf of the Editorial Board of the *International Journal of Molecular Sciences*, we would like to congratulate the winning teams for their excellent work. In recognition of their accomplishments, they will receive the privilege of publishing an additional research article or review paper free of charge in open access format in the *International Journal of Molecular Sciences*, after the usual peer-review procedure.

We would like to take this opportunity to thank all the nominated research groups of the above exceptional papers for their contributions to the *International Journal of Molecular Sciences*, and thank the Editorial Board of the *International Journal of Molecular Sciences* for assessing and helping with the selection of this “Best Paper Award”.

The Editorial Board and Editorial Staff at the *International Journal of Molecular Sciences* are committed to meeting the needs of the molecular research community by providing extensive and timely reviews of all submitted manuscripts, and by providing an open access forum for the publication of your results. Please consider submitting your work to the *International Journal of Molecular Sciences*, and we look forward to announcing your manuscript as a winner of the *International Journal of Molecular Sciences* Best Paper award in the future.

## Prize Awarding Committee

The Editorial Board of the *International Journal of Molecular Sciences*
